# Structure and diversity of mycorrhizal fungi communities of different part of *Bulbophyllum tianguii* in three terrestrial environments

**DOI:** 10.3389/fpls.2022.992184

**Published:** 2022-10-05

**Authors:** Jiayu Liang, Rong Zou, Yang Huang, Huizhen Qin, Jianmin Tang, Xiao Wei, Yu Liang, Shengfeng Chai

**Affiliations:** ^1^ Key Laboratory of Ecology of Rare and Endangered Species and Environmental Protection, (Guangxi Normal University), Ministry of Education, Guilin, China; ^2^ Guangxi Key Laboratory of Landscape Resources Conservation and Sustainable Utilization in Lijiang River Basin, College of Life Science, Guangxi Normal University, Guilin, China; ^3^ Guangxi Key Laboratory of Plant Functional Phytochemicals and Sustainable Utilization, Guangxi Institute of Botany, the Chinese Academy of Sciences, Guilin, China; ^4^ School of Mechanical and Electrical Engineering, Guilin University of Electronic Technology, Guilin, China

**Keywords:** orchidaceae, mycorrhizal fungi, *bulbophyllum tianguii*, microbial community, orchids

## Abstract

Mycorrhizal fungi plays important roles in the seed germination and subsequent growth of orchids. The research of fungi in orchid roots, especially dominant mycorrhizal fungi is critical for orchids protection. In this study, the fungal community and composition of mycorrhizal fungi in roots, rhizomes and rhizosphere soil of *Bulbophyllum tianguii* grown in three terrestrial environments were analyzed by the second generation sequencing technology. The results of OTU clustering and α and β diversity analysis showed that there were significant differences in fungal communities in roots, rhizomes and rhizosphere soil of *B. tianguii*. The total number of OTUs in rhizomes was much less than that in roots and rhizosphere soil. The number of OTUs in rhizosphere soil and the diversity of mycorrhizal fungi were the highest. Meanwhile, the species and abundance of mycorrhizal fungi in roots and rhizomes of *B. tianguii* were different from those in rhizosphere soil. For different elevations, compared with *B. tianguii* that grow in middle of Tiankeng and top of Tiankeng, the OTUs number of *B. tianguii* in orchid garden is richest, and the diversity of mycorrhizal fungi in orchid garden was significantly higher than other locations. Among the three different habitats of *B. tianguii*, the number of OTUs in humus soil and stone habitats was notably higher than tree habitats, and the diversity of mycorrhizal fungi in humus soil was the highest. The analysis of mycorrhizal fungi in different habitats and altitudes of *B. tianguii* showed that Sebacina and Exophiala were the dominant mycorrhizal fungi in *B. tianguii*. The results of species annotation, phylogenetic tree and co-occurrence network analysis showed the dominant mycorrhizal fungi of *B. tianguii* mainly included Sebacina, Cladosporium, Exophiala, Fusarium. This study reveals the symbiotic relationship between Sebacina, Exophiala, Cladosporium and the *B. Tianguii*. It will provide a theoretical basis for the protection and biological function study of *B. Tianguii*.

## Introduction

Orchidaceae, commonly known as Orchids, is one of the most abundant and diverse groups in the plant kingdom, widely distributed in various terrestrial ecosystems with a long evolutionary history (Li T. et al., 2021; Wang et al., 2021). Due to the important ornamental value, medicinal value and cultural value of orchids, the phenomenon of excessive excavation of orchids is hardly avoided, resulting in almost all wild orchids in different degrees of endangerment (Pandey et al., 2013). The Convention on International Trade in Endangered Species of Wild Fauna and Flora (CITES) includes all wild orchids in appendices I and II, and more than 90% of the protected plants covered by CITES are orchids.

*Bulbophyllum tianguii* is a new species of orchidaceae which was found in Guangxi Yachang orchidaceae National Nature Reserve in 2007 (Lang and Luo, 2007). It is mainly distributed on the surface of karst stone mountains or on the stems of humus trees. This species was only found in four distribution sites in Yachang Reserve, and was subsequently found in Guangxi Mulun National Nature Reserve, Guizhou Wangmo Sutie Nature Reserve (Luo et al., 2020). The associated plants include trees, shrubs, vines, herbs, and orchids: *Eriacoronaria*, *Cymbidiumfloribundum*. As one of the star orchids in Yachang Reserve, the related researches about *B. tianguii* is little. Only one biological study on the pollination mechanism of *B. tianguii* in Laowuji Tiankeng, Yachang Reserve is reported. This study showed that the seed setting rate of *B. tianguii* was very low under the natural state, and the seed setting rate of *B. tianguii* could be significantly increased to 100% by pollinating by crushing pollen (Jiang et al., 2020). Therefore, the further study of mycorrhizal fungi in roots and rhizosphere soil can provide effective measures for the conservation of *B. tianguii*. Orchids growing at different altitudes will be affected by the gradual decrease of temperature, humidity, nitrogen (N), phosphorus (P) and potassium (K) contents in soil, and orchids will improve their adaptability to the environment, which will in turn affect the number and species of mycorrhizal fungi to a certain extent. At the same time, mycorrhizal fungi could promote the absorption of N, P, K, Ca, Mg and other elements (Chen et al., 2017; Ren et al., 2021).

The survival and growth of orchids are highly depend on the symbiotic fungi in their roots, among which mycorrhizal fungi is important in the seed germination and subsequent growth of orchids (Hou et al., 2010; Tian et al., 2022). Research on mycorrhizal fungi in orchid roots has significance in the protection of orchids. Mycorrhizal fungi, also known as symbiotic fungi, are a group of fungi that can form a specific symbiotic structure with the roots of higher plants or other root-like organs in contact with the substrate. They live on the surface, in the cortex, or around the epidermal cells of roots (root-like organs) and are beneficial to both plants and fungi (Wang et al., 2021). The study of mycorrhizal fungal community of *Spiranthes sinensis* found that there were significant differences in the composition of the mycorrhizal diversity of the 6 samples of *Spiranthes sinensis*, and there was no obvious correlation between the differences and geographical distance, suggesting that the composition of the mycorrhizal community was more affected by habitat factors (Li J. et al., 2021). Studies about *Cypripedium* species at different altitudes found that the community structure of mycorrhizal fungi was significantly different among three *Cypripedium* species, indicating that the preference of *Cypripedium* to mycorrhizal fungi significantly affected the community structure of mycorrhizal fungi in the same habitat (Xu et al., 2019).

The root system of plants is critical in the whole life activities of plants, mainly absorbing water and elements in the soil. Plant roots can provide sufficient energy and nutrition for various microorganisms in the rhizosphere by secreting rich organic substances into the soil, and regulating the types and numbers of these microorganisms (Wu et al., 2014). At the same time, the detection of mycorrhizal fungi in the soil can determine whether the site is suitable for the orchids growth (Li T. et al., 2021). Therefore, studying the distribution of mycorrhizal fungi in the roots of orchids in the surrounding soil is important in the population restoration of orchids. Some studies about mycorrhizal fungi in orchid roots and the surrounding soil are reported. The results show that most of the mycorrhizal fungi found in roots are also widely distributed in soil (Waud et al., 2016). At the same time, other studies found that the mycorrhizal fungi communities in orchid roots and rhizosphere soils are significantly different (Liu et al., 2015; Han et al., 2016). The second generation sequencing technology was used to analyze fungal communities and mycorrhizal fungi composition in roots, rhizosphere soil and rhizosphere soil of nine orchids belonging to minimal populations in Liaoning Province, China. It was also found that the OTUs in roots of orchids were less than that in rhizosphere soil and the bulk soil. The species and abundance of mycorrhizal fungi in orchid roots were not significantly correlated with that in the rhizosphere soil and the bulk soil, indicating that the fungal communities in orchid roots and soil were independent to some extent (Jiang et al., 2019).

The relationship between the community structure and habitat of symbiotic mycorrhizal fungi in the same orchids is one of the important contents in the study of both orchids and mycorrhizal fungi. Some studies have proved that the mycorrhizal fungi communities of the same orchids have great variability, which may vary under different habitats. Environmental conditions can strongly affect the mycorrhizal fungi communities of orchids (Esposito et al., 2016; Li T. et al., 2021). On the other hand, microbial communities in soil are influenced not only by rhizosphere secretions but also by many other factors, such as climate and the physical and chemical properties of the soil (Kaushik et al., 2021). The changes of mycorrhizal fungi communities in the same orchids in different habitats may be related to the physical and chemical properties of local soil. By analyzing the diversity of endophytic fungi in *Cremastra appendiculata* roots and exploring the effects of altitude and rhizosphere soil physical and chemical properties on fungal diversity, it was found that the diversity of fungi varied in different elevations. Nitrate nitrogen and available phosphorus in rhizosphere soil decreased with elevation. The higher the altitude, the less endophytic fungi. And the more uneven the community distribution leads to the more prominent the dominant fungi (Ren et al., 2021). In the study of mycorrhizal fungi frequency on the volcano Mount Koma in northern Japan, it was found that ammonium nitrogen content at the lowest altitude was more than twice as high as that at the highest altitude. The nitrate content also fluctuated with altitude, but the difference was not significant except the lowest at 750 m altitude. With the increase of altitude, nitrogen and phosphorus levels decreased, indicating that higher altitude may have a certain impact on the survival of mycorrhizal fungi and their host environmental adaptation (Tsuyuzaki et al., 2005). While comparing the fungal community composition of *Gymnadenia conopsea* in southern Tibet (3600 m) and northern Heilongjiang Province (496 m), it was found that Ambisporaceae, Archaeosporaceae, Acaulosporaceae, Gigasporaceae, Glomeraceae and Paraglomeraceae were significantly enriched in the soil of southern Tibet (3600 m), while these fungi were almost not found in northern Heilongjiang Province (496 m). In addition, Clavariaceae, Russulaceae, Leotiaceae and Dictyosporiaceae are dominant in the roots of southern Tibet (3600 m), while these families are rare in the roots of northern Heilongjiang Province (496 m). The low pressure, low oxygen and strong radiation in high altitude areas will have adverse effects on the growth of plants. The above research results show that these fungal groups may play a role in *G. conopsea*’s adaptation to the high altitude environment in southern Tibet (Lin et al., 2020). It can be seen from the above studies that the increase of altitude will be accompanied by the decrease of air temperature and pressure, and the increase of photosynthetic radiation. Whether orchid mycorrhizal fungi or their hosts, they will have corresponding adaptation to environmental changes, as well as the interaction between microorganisms and plants, which may cause differences in fungal community composition. Other studies showed that there was specificity between mycorrhizal fungi and orchids. It was found that there were commonness and difference in the dominant population of wild orchid mycorrhizal fungi with different geographical distribution. The mycorrhizal fungi of *C. goeringii* in Dongbu Mountain were more diverse and distributed more evenly. Meanwhile, due to the influence of regional habitat, the diversity, good conditions and rich mycorrhizal fungi are conducive to the growth of *Cymbidium goeringiensis* (Xu and Liu, 2020). These results suggest that the changes of orchidaceae mycorrhizal fungi in different habitats may be related to the characteristics of plants themselves, but the effect of habitats on orchidaceae mycorrhizal fungi needs further research.

*B. tianguii* is an epiphytic plant of the orchid family. Its roots can attach to humus, rocks and tree trunks. The root mycorrhizal fungi and the root and rhizosphere soil mycorrhizal fungi may be different in the three habitats. Different altitudes affect the adaptability of *B. tianguii*, and the species and quantity of mycorrhizal fungi may change. The present study analyze the similarities and differences of mycorrhizal fungi communities in roots and rhizosphere soil of *B. tianguii* in different habitats, and find out the most important mycorrhizal fungi species, which can provide significant basis for the conservation and biological research of orchid plants.

## Materials and methods

### Plant material and soil sampling

**Figure 1:**
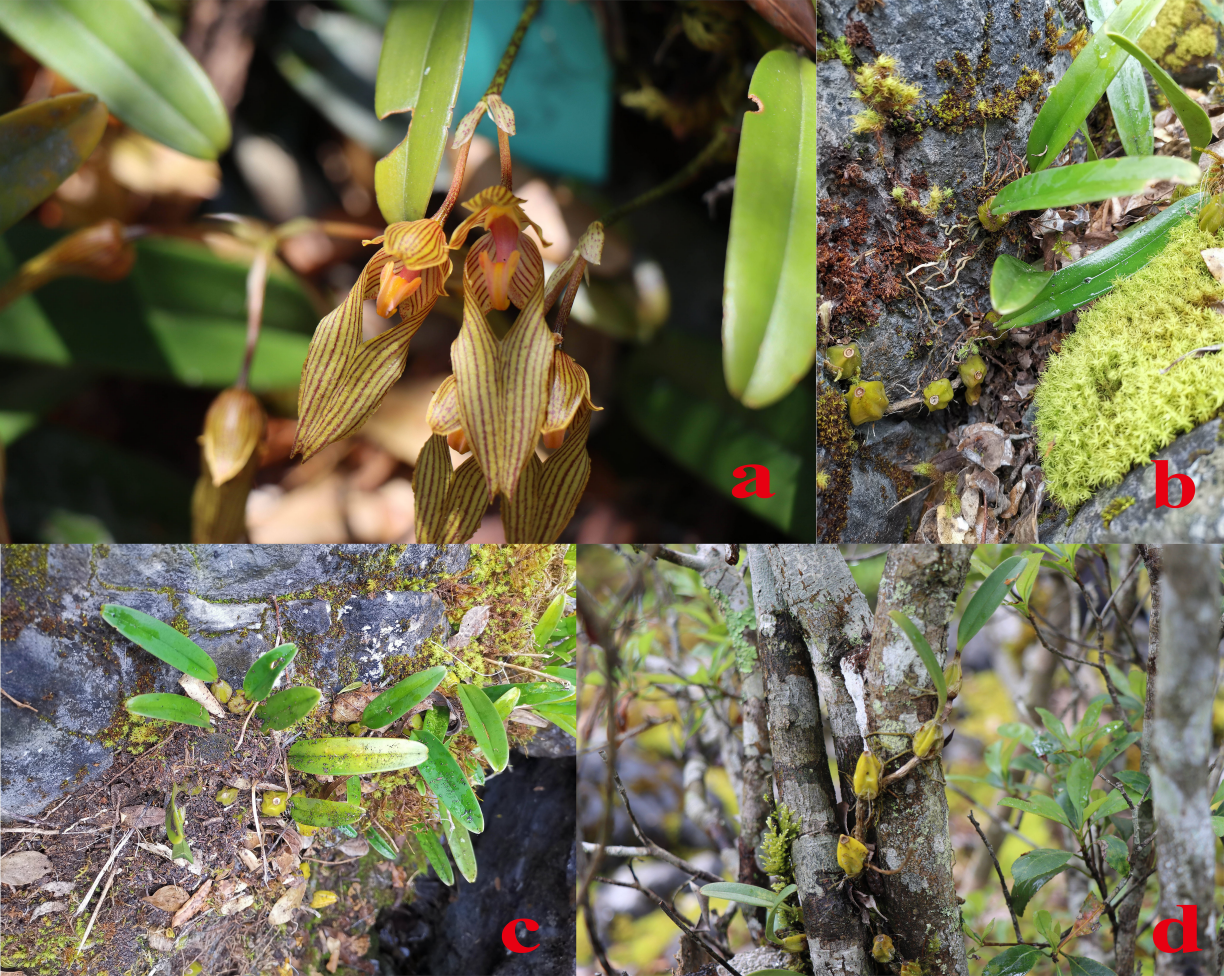
Growth situation of *B tianguii*. [**(A)**: *B tianguii*; **(B)**: Roots attached to stone walls; **(C)**: Roots attached to humus soil; **(D)**: Roots attached to the trunk.].

The roots, rhizomes and rhizosphere soil (bark from trees and moss from rocks) of *B. tianguii* were collected from three populations at different elevations in Yachang Orchidaceae National Nature Reserve, Guangxi province. The three main habitats of *B. tianguii* are humus soil, stones and trees. The different samples from all these three habitats were collected. In order to investigate the influence of altitudes for *B. tianguii*, the samples from three different altitudes (middle of Tiankeng, top of Tiankeng and orchid garden) were also collected. The fungal species of each part were determined with 3 biological replicates and a total of 81 samples were collected. The distribution habitat (**Figure 1**) and population situation and details of *B. tianguii* samples are shown in **Table 1**. The collected root segment and environmental samples were stored in dry ice and brought back to the laboratory for subsequent nucleic acid extraction experiments.

**Table 1:** Habitat and population profile of *Bulbophyllum tianguii*.

Plot	Site	Altitude (m)	Slope (°)	Aspect	Slope position	Distribution area (m^2^)	Number of pseudobul	Number of stems	Density of dense distribution area (Numberof stems/m^2^)
P1	Orchid Garden	950	30	Northwest	The upper part of Shishan Mountain	12	1270	125	35
P2	Middle of Tiankeng	1198	80	Southwest	The top of Tiankeng	35	2039	200	32
P3	Top of Tiankeng	1281	15	West	Near the mountain top	15	4360	427	82

### DNA extraction and sequencing

E.Z.N.A.^®^ Soil DNA Kit was used to extract total microbiome DNA from all samples, and 1% agarose gel electrophoresis was used to detect extracted genomic DNA. Specific primers with barcode were synthesized according to the designated sequencing area. PCR amplification primers corresponding to the region of ITS1F_ITS2R (Lemons et al., 2017) the upstream primer ITS1F: CTTGGTCATTTAGAGGAAGTAA, and the downstream primer ITS2R: GCTGCGTTCTTCATCGATGC. PCR amplification was performed using TransGen AP221-02: TransStart Fastpfu DNA Polymerase and PCR instrument: ABI GeneAmp^®^ 9700. Reaction PCR reaction system includes: 4 µL5×FastPfu Buffer; 2 µL2.5 mM dNTPs; 0.8 µL upstream primer (5 µM); 0.8 µL downstream primer (5 µM); 0.4 µL FastPfu DNA Polymerase; 0.2 µL BSA; 10 ng genome DNA; supply ddH_2_O to 20 µL. The PCR amplification cycle in this study was pre-denaturation at 95°C for 3 min, 30 cycles: denaturation at 95°C for 30 s, annealing at 56°C for 30 s, extension at 72°C for 45 s, and finally stable extension at 72°C for 10 min (Xu et al., 2019). Each sample had three replicates. The PCR products of the same sample were mixed and detected by 2% agarose gel electrophoresis. The products were eluted with Tris_HCl reagent, and the PCR products were recovered by gel cutting with AxyPrepDNA gel recovery kit (AXYGEN Company). The PCR products were detected and quantified using the QuantiFluor™ -ST blue fluorescence quantification system (Promega Company) based on the preliminary quantitative results of electrophoresis, and the products were mixed in corresponding proportions according to the sequencing requirements of each sample. Purified amplified fragments were used to construct Miseq library using NEXTFLEX Rapid DNA-Seq Kit and sequenced using Illumina Miseq PE300 platform (Shanghai Meiji Biomedical Technology Co., Ltd.). FASTP (Chen et al., 2018) (https://github.com/OpenGene/fastp,version 0.20.0) software was used to control the quality of the original sequencing sequences of all samples. According to the overlap relationship between PE reads, pairs of reads were spliced into a sequence. The minimum overlap length was 10 bp, and the maximum mismatch ratio allowed in the overlap region of the spliced sequence was 0.2, which was used to screen sequences that did not meet the requirements of this experiment. Use FLASH (Magoc and Salzberg, 2011)(http://www.cbcb.umd.edu/software/flash,version 1.2.7) software for quality control sequence stitching.

### Data analysis

#### OTU analysis

In order to facilitate the analysis of the species and number of fungi in the sequencing results, UPARSE software (Version 7.1) (Edgar, 2013) was used to cluster the optimized sequences and they were divided into groups according to the similarity between sequences (97%). A group is an Operational Taxonomic unit (OTUs) (He et al., 2015). The representative sequences of OTUs were taxonomic analyzed and summarized by RDP classifier (similarity > 97%) to obtain the classification information related to each OTUs (Wang et al., 2007), and the microbial community composition of each sample was counted at each classification level. In this study, Unite8.0/ITS_Fungi classification database was used for taxonomic analysis of OTUs.

#### Rarefaction curve analysis

The Rarefaction curve (Bacaro et al., 2012) is constructed by randomly selecting the result sequence from sequencing and combining the sequence number with the corresponding species number or diversity index. Mothur (Schloss et al., 2009) was used to analyze various Alpha diversity indexes, and R language was used to make dilution curves of each sample, so as to analyze whether the sample sequencing depth was sufficient. Dilution curves can also be used to compare species richness, uniformity or diversity among samples with different data volumes.

#### Community composition analysis

Through the amount of sample data in overlapping and separated areas in the Venn diagram, the number of common and unique OTUs in each sample was shown, so as to directly observe the similarity and overlap of OTUs number composition among each sample, as well as the number of unique groups in each sample’s microbial community. And through the community Bar diagram to analyze the fungal community at all levels of classification groups and dominant species.

#### Alpha-diversity analysis

Alpha diversity is used to explore the diversity of microbial communities within the sample and the richness and evenness between communities by means of a variety of statistical indices (Baczkowski et al., 1998). Good’s Coverage index is mainly used to reflect community coverage, while sobs, chao1 and Ace (Adaptive coherence estimator) can reflect the community richness of microbial communities. Shannon and Simpson index can show the community diversity of microbial community. The samples were divided into groups, and the Wilxocon rank sum test was used to analyze the differences between groups of Alpha diversity, and to detect whether the index values between the two groups had significant differences.

#### Beta-diversity analysis: NMDS analysis

NMDS (non-metric multidimensional scaling) analysis is used to simplify the research objects (samples or variables) of multidimensional space to low-dimensional space for positioning, analysis and classification. At the same time, it can retain the original relationship between objects data analysis method. According to the species information contained in the samples, it is reflected in the multidimensional space in the form of points, and the degree of difference between different samples is reflected by the distance between points, and finally the spatial location map of the samples is obtained. Using Qiime to calculate beta diversity distance matrix, R language (Version 3.3.1) vegan software package for NMDS analysis and mapping.

#### Phylogenetic tree analysis

Phylogenetic trees were constructed according to the evolutionary relationships among microbial species in *B. tianguii*, and the phylogenetic relationships among species in the samples were revealed from the perspective of molecular evolution. Through the software: FastTree (version 2.1.3 http://www.microbesonline.org/fasttree/). By selecting the sequence corresponding to the classification information at the OTU level, the evolutionary tree is constructed according to Maximum Likelihood (ML), and the evolutionary tree is plotted using R language (Version 3.3.1).

#### Co-occurrence network analysis

Co-occurrence network analysis was used to display samples and species distribution. By analyzing the species abundance information between different environmental samples, the co-existence relationship of species in environmental samples can be obtained to highlight the similarities and differences between samples. Co-occurrence network analysis was performed by Networkx software.

## Results and analysis

### Fungal composition in roots and rhizosphere soil of *B. tianguii*

In the present study, a total of 81 samples were collected from 27 root samples, 27 rhizome samples and 27 rhizosphere soil samples of *B. tianguii*, which grow in Guangxi Yachang orchidaceae National Nature Reserve (**Table 1**). These 81 samples of *B. tianguii* were collected from three different growth environments of humus soil, stone and tree in three different altitude sites: middle of Tiankeng, top of Tiankeng and orchid garden (**Figure 1**). The different habitats and altitudes may affect the number and species of mycorrhizal fungi in orchid roots and rhizosphere soil. At different altitudes, the number of possible mycorrhizal fungi could be different; similarly, in different habitats, the *B. tianguii* grown in humus soil habitat may have more mycorrhizal fungi than stones and trees, speculated that humus soil may be moist and rich in nutrients suitable for fungal growth. After Illumina MiSeq high-throughput sequencing, quality control splicing of double-end sequences and OTU clustering, a total of 4447492 sequences were obtained, including 1667918 for roots, 1379856 for rhizomes and 1399718 for rhizosphere soil. A total of 6051 OTUs were obtained from root samples, 3482 OTUs from rhizome, and 5560 OTUs from rhizosphere soil. The common and unique fungal OTUs of *B. tianguii* samples were expressed as the Venn diagram to evaluate the relationship between fungal communities in roots, rhizomes and rhizosphere soils of *B. tianguii* at different habitats and altitudes. There are 2514 OTUs were shared by root, rhizome and rhizosphere soil; 293 OTUs were shared by root and rhizome; 331 OTUs were shared by rhizome and rhizosphere soil; and 2266 OTUs were shared by root and rhizosphere soil. The numbers of OTUs unique to each species were as follows: 978 for root, 344 for rhizome, and 1643 for rhizosphere soil. Except for non-annotated fungi, the distribution of fungi in the root, rhizome and rhizosphere soil of *B. tianguii* at different classification levels among domain to species is shown in **Table 2**. The number of OTUs of mycorrhizal fungi in B.tianguii samples varied in different habitats and altitudes. Overall, the number of OTUs of mycorrhizal fungi in the roots, rhizomes and rhizosphere soil of B.tianguii in humus soil habitat and orchid garden was more abundant. On phylum classification level, Ascomycota, Basidiomycota, Rozellomycota, Mortierellomycota, Chytridiomycota, Glomeromycota, Mucoromycota, unclassified_k:Fungi are the common phylums of the fungi detected in roots, rhizomes and rhizosphere soil samples of *B. tianguii*. In addition, the fungi in root also include Kickxellomycota, and the fungi in rhizosphere soil also includes Kickxellomycota, Basidiobolomycota, Neocallimastigomycota, Olpidiomycota.

**Table 2:** OTU statistics of each classification level (Fungi).

Sample name	Domain	Kingdom	Phylum	Class	Order	Family	Genus	Species
Root	1	1	9	40	126	344	880	1511
Rhizome	1	1	8	32	104	286	663	1053
Rhizosphere soil	1	1	12	53	141	354	860	1429

It can be seen from the above results that the number of fungi in the root and rhizosphere soil of *B. tianguii* was apparently higher than that in the rhizome, and the species of fungi in the rhizosphere soil was the richest. The number of effective sequences in 81 samples was randomly selected by Mothur analysis, and the dilution curve (**Supplementary Figure S1**) was drawn by sobs index. It can be seen that the curve began to flatten after exceeding 25000, indicating that the sequencing has reached saturation and the most OTUs have been obtained.

### Mycorrhizal fungi composition of *B. tianguii*

**Figure 2:**
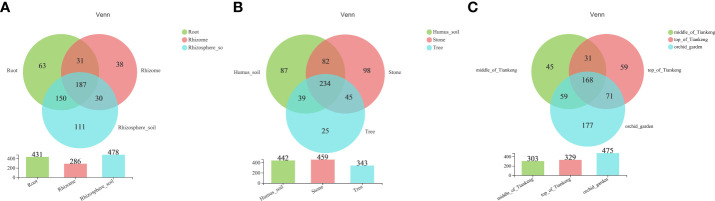
Venn chart. Different colors represent different groups, the number of overlapping parts represents the number of species shared by multiple groups, and the number of non-overlapping parts represents the number of species unique to the corresponding group. [**(A)**: root, rhizome and rhizosphere soil groups; **(B)**: different habitat groups; **(C)**: different altitude groups].

According to the published list of common orchid mycorrhizal fungi summarized by Wang et al. (2021), mycorrhizal fungi were selected from all the fungi detected in 81 samples of *B. tianguii*. A total of 209, 162 and 215 fungi were detected from root, rhizome and rhizosphere soil, respectively (**Table 3**), all belonging to the following four phylums: Ascomycota, Basidiomycota, Mucoromycota and Mortierellomycota. It can be seen that the OTUs in rhizosphere soil of *B. tianguii* are more than those in root, but the number at each classification level is basically the same. And the OTUs of rhizome are far less than those of root and rhizosphere soil. There are 187 common OTUs in root, rhizome and rhizosphere soil; 31 OTUs were shared by root and rhizome; 30 OTUs were shared by rhizome and rhizosphere soil; and 150 OTUs were shared by root and rhizosphere soil. The numbers of OTUs unique to root, rhizome and rhizosphere soil were 63, 38 and 111, respectively (**Figure 2A**). The results show that the number of mycorrhizal fungi in rhizosphere soil of *B. tianguii* was the highest, which was much higher than that of the rhizome. It is known that the roots of *B. tianguii* are closer to soil while the rhizomes are farther. The number of mycorrhizal fungi in the roots of is close to that in the rhizosphere soil, and both are significantly higher than rhizomes. There were 234 species of mycorrhizal fungi in the samples collected from humus soil, stone and tree habitats. The number of mycorrhizal fungi in humus soil habitats was 87, and 96 in stone habitats, and 25 in tree habitats. The results showed that the number of mycorrhizal fungi in the root endophyte and rhizosphere soil of *B. tianguii* grown on the tree was significantly lower than that in the humus soil and stone habitats. There are 168 OTUs were shared by the middle of Tiankeng, the top of Tiankeng and the orchid garden; 31 OTUs were shared by the middle of Tiankeng and the top of Tiankeng; 71 OTUs were shared by the top of Tiankeng and the orchid garden; and 59 OTUs were shared by root and rhizosphere soil. The numbers of OTUs unique to the middle of Tiankeng, the top of Tiankeng and the orchid gardenwere as follows: 45 for the middle of Tiankeng, 59 for the top of Tiankeng, 117 for orchid garden (**Figures 2B, C** **)**. The number of mycorrhizal fungi in the orchid garden population was significantly higher than that in the two places, which was consistent with the results of fungal statistics.

**Table 3:** OTU statistics of each classification level (Mycorrhizal fungi).

Sample name	OTUs	Domain	Kingdom	Phylum	Class	Order	Family	Genus	Species
Root	431	1	1	4	9	21	35	52	209
Rhizome	286	1	1	4	9	21	33	49	162
Rhizosphere soil	478	1	1	4	8	20	34	52	215

**Figure 3:**
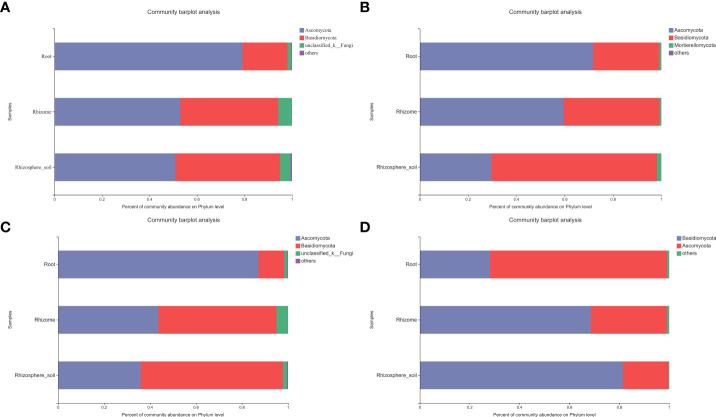
Bar diagram of fungal community composition. The ordinate is the sample name, and the abscissa is the proportion of species in the sample. Columns with different colors represent different species, and the length of the column represents the proportion of the species. **(A)**: fungi composition at Phylum level; **(B)**: mycorrhizal fungi composition at Phylum level; **(C)**: fungal composition of the middle of Tiankeng (Phylum); **(D)**: mycorrhizal fungi composition of the middle of Tiankeng (Phylum).

In order to identify the fungal taxa related to *B. tianguii* and analyze their similarities and differences in root and rhizosphere soil of *B. tianguii* under different habitats, especially the distribution of mycorrhizal fungi. Two taxonomic levels (phylum and genus) were selected for further analysis (**Figures 3A, B** **)**. Among the fungal taxa of *B. tianguii*, Ascomycota and Basidiomycota were the most representative phylum. In most samples, Ascomycota dominated (79.16%-50.96%), and Ascomycota was particularly abundant in root samples (79.16%). The mycorrhizal fungi belonging to Ascomycota were dominant in the roots and rhizomes of *B. tianguii* (71.75% and 59.52%), and the dominant group in rhizosphere soil was Basidiomycota (68.56%). The results showed that Ascomycota fungi were the main endophytic fungi of *B. tianguii*, and Basidiomycota fungi were the main exogenous fungi, indicating that there were significant differences between endophytic fungi and exogenous fungi in *B. tianguii*. The fungal classification at different sites showed great differences in the phylum classification level of fungi and mycorrhizal fungi in the sites with different elevation. At the same time, the main dominant groups of *B. tianguii* fungal community in each taxonomic unit (phylum to genus) were also sorted out (**Supplementary Table 4**). Notably, Ascomycota (87.16%) was the dominant phylum in the fungal community of root samples collected in the middle of Tiankeng. And Basidiomycota (51.34%/61.86%) was the dominant phylum in rhizomes and rhizosphere soil (**Figure 3C**). In the mycorrhizal fungal community of root samples collected in the middle of Tiankeng, Ascomycota (71.02%) was the dominant phylum, and Basidiomycota (68.54%/81.45%) was the dominant phylum in rhizome and rhizosphere soil (**Figure 3D**). The dominant groups of *B. tianguii* fungi and mycorrhizal fungi in the middle of Tiankeng are different from the overall situation, which maybe related to the environment in the middle of Tiankeng and the adaptability of orchids to this altitude.

**Figure 4:**
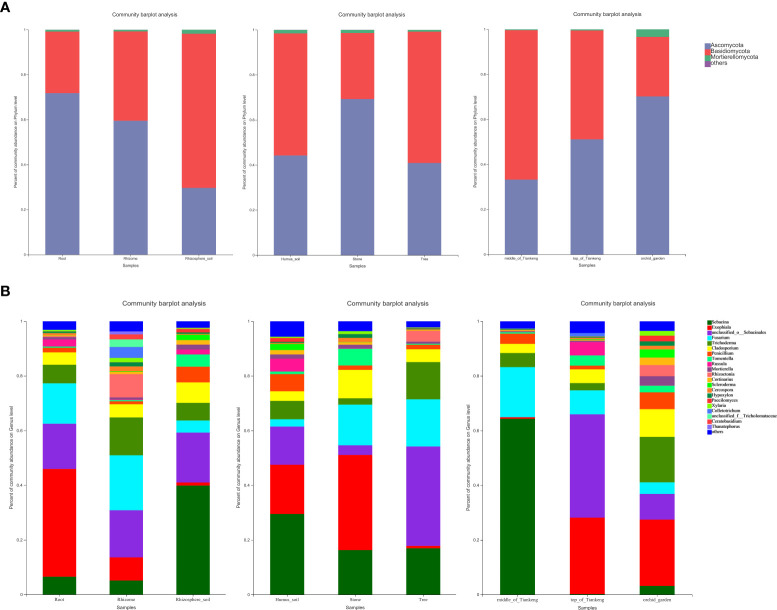
Bar diagram of mycorrhizal fungal community composition. The abscissa is the sample name, and the ordinate is the proportion of species in the sample. Columns with different colors represent different species, and the length of the column represents the proportion of the species. Composition of mycorrhizal fungi is at Phylum **(A)** and Genus levels **(B)**.

According to the genera of some common orchid mycorrhizal fungi published by Wang et al. (2021), mycorrhizal fungi were screened from all fungi detected in all 81 samples. In addition to unclassified _o:Sebacinales, combined with the main dominant groups of mycorrhizal fungi in each taxonomic unit (**Supplementary Table 5**), it can be seen that the species of endophytic mycorrhizal fungi, rhizome mycorrhizal fungi and rhizosphere soil mycorrhizal fungi in *B. tianguii* are basically similar to those of endophytic mycorrhizal fungi at the phylum to genus levels, but there are great differences in the species and abundance of rhizosphere soil mycorrhizal fungi (**Figure 4**). And the main dominant groups also have significant differences. The dominant genus of endophytic fungi in *B. tianguii* is Exophiala (39.43%), followed by Fusarium (14.74%). The dominant genus of endophytic fungi in rhizomes of *B. tianguii* is Fusarium (20.13%), and the dominant species is Fusarium_concentricum (18.85%). It can be seen that the dominant mycorrhizal fungi in the roots and rhizomes were Exophiala and Fusarium, while the dominant mycorrhizal fungi in the rhizosphere soil of *B. tianguii* were Sebacina (39.87%), and the dominant species were Sebacina_ sp. (39.85%). The results showed that *B. tianguii* chose Exophiala and Fusarium as its endogenous dominant mycorrhizal fungi. And Sebacina that function as its exogenous dominant mycorrhizal fungi was more conducive to the growth and reproduction of orchids in rhizosphere soil. Among the *B. tianguii* samples in different habitats (humus soil, stone and tree), mycorrhizal fungi belonging to Ascomycota were dominant in stone habitat (69.27%), and the dominant groups in humus soil and tree habitat were Basidiomycota (54.19% and 58.30%). At genus level, the dominant mycorrhizal fungi in humus habitat are Sebacina (29.48%), Exophiala (34.79%) in stone habitat, and unclassified in tree habitat_o:Sebacinales(36.36%). Among the *B. tianguii* samples from different altitudes (middle of Tiankeng, top of Tiankeng and orchid garden), mycorrhizal fungi belonging to Ascomycota were dominant in the orchid garden population (70.18%), and the dominant groups of middle of Tiankeng and top of Tiankeng populations were Basidiomycota (66.38% and 51.14%). At genus level, the dominant mycorrhizal fungi in the middle population of Tiankeng are Sebacina (64.30%), and the dominant mycorrhizal fungi in the top population of Tiankeng are unclassified_ o: Sebacinales (37.79%), and Exophiala (24.23%) was the dominant mycorrhizal fungus in Orchid Garden population.

### Alpha-diversity analysis

**Figure 5:**
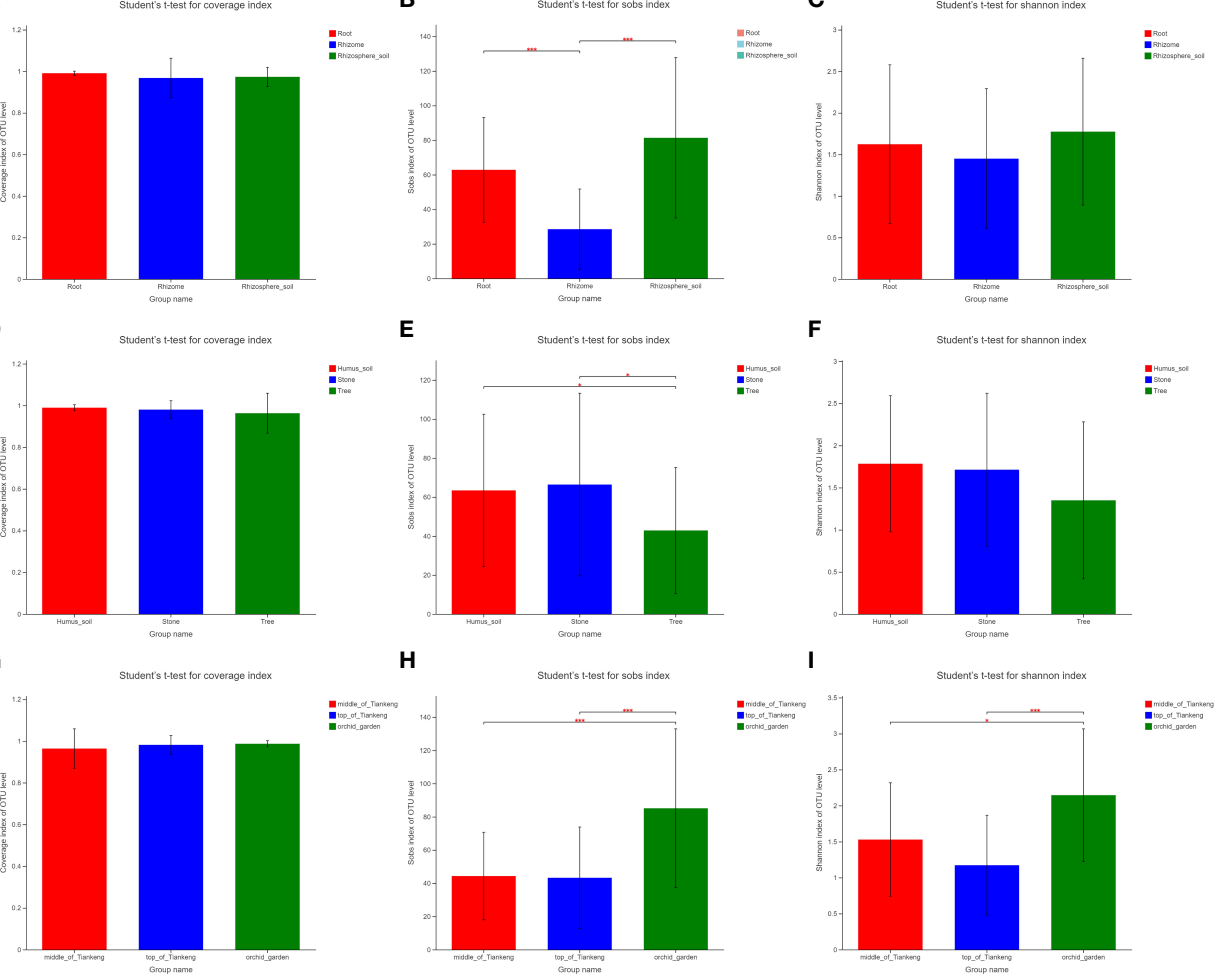
Wilxocon rank sum test of Alpha index difference between groups. The significance of the difference between the two groups is expressed as: 0.01 <p ≤ 0.05 marked for *, P ≤ 0.001 marked for ***. The abscissa is the grouping name, and the ordinate is the index average of each group. **(A-C)**: root, rhizome and rhizosphere soil groups; **(D–F)**: different habitat groups; **(G–I)**: different altitude groups.

Based on the results of t-test of the difference between groups of alpha diversity index, this paper selects sobs, Shannon and coverage to analyze the diversity of *B. tianguii* fungal community. According to the grouping analysis of the sample type (root, rhizome and rhizosphere soil) of *B. tianguii*, the diversity index coverage results are higher than 0.95, and the difference between groups is not significant, indicating that the sequencing results represent the real situation of microorganisms in each group of samples (**Figures 5A, D, G** **)**. The t-test of inter group difference of sobs index showed that the number of mycorrhizal fungi OTUs observed in rhizome was significantly lower than that in root and rhizosphere soil samples (P ≤ 0.001, marked as * * *), the difference of OTUs observed in root and rhizosphere soil samples was not significant, and the number of mycorrhizal fungi in rhizosphere soil was slightly higher than that in root (**Figure 5B**). The t-test results of the inter group difference of Shannon index show that there is no significant difference in the community diversity of mycorrhizal fungi among roots, rhizomes and rhizosphere soil (**Figure 5C**), which is consistent with the species annotation results in the previous classifications. The order of diversity is rhizosphere soil > root > rhizome. The results showed that the mycorrhizal fungi in *B. tianguii* rhizosphere soil were the most abundant. According to different altitude populations of *B. tianguii* (middle of Tiankeng, top of the Tiankeng, orchid garden), T test of Sobs index showed that the number of fungal OTUs observed in samples collected at orchid garden was higher than that at middle of Tiankeng and top of Tiankeng, and the difference was extremely significant (P ≤ 0.001 marked as * * *). There is no significant difference between the middle of Tiankeng and the top of Tiankeng (**Figure 5E**). The T-test results of the inter-group difference of the shannon index (**Figure 5F**) indicated that the mycorrhizal fungal community diversity of the samples from the orchid garden was significantly higher than that of the top of Tiankeng (0.001 < P ≤ 0.01 marked as * *). This indicated that the mycorrhizal fungi in the roots and rhizosphere soil of *B. tianguii* grown in orchid gardens were the most abundant. According to the grouping analysis of different habitats (humus, stone, and tree) of *B. tianguii*, the T test results of the difference between groups of Sobs index (**Figure 5H**) indicated that the number of mycorrhizal fungi OTUs observed in the samples collected in the tree habitat was significantly lower than that in the humus and stone habitats (0.01 < P ≤ 0.05 marked as *), while there was no significant difference in the number of mycorrhizal fungi OTUs between the humus and stone habitats. The shannon index of each group of *B. tianguii* on humus soil, stone and tree ranked as humus soil > stone > tree (**Figure 5I**). The number and species of mycorrhizal fungi of *Bulbophyllum tianguii* growing in humus soil and stone habitat are more abundant, indicating that the mycorrhiza fungi of *B. tianguii* are more abundant in humus soil, which may be more suitable for *B. tianguii* habitat.

### Beta-diversity analysis: NMDS analysis

**Figure 6:**
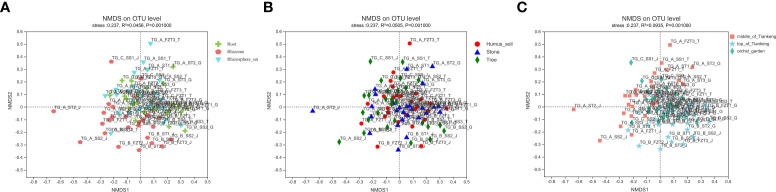
NMDS analysis. Points of different colors or shapes represent samples of different groups. The closer the two sample points are, the more similar the species composition is. **(A)**: root, rhizome and rhizosphere soil groups; **(B)**: different habitat groups; **(C)**: different altitude groups.

NMDS analysis (**Figure 6**) showed that the order of sample points in root and rhizosphere soil was basically concentrated, while the order of sample points in rhizome was relatively dispersed, and the order of sample points in humus soil, stone and tree was also relatively dispersed. The order of sample points in the middle and top of the Tiankeng was relatively concentrated, but the order of sample points in orchid garden was relatively dispersed. However, there was no obvious separation among the groups of samples, indicating that there were some differences in mycorrhizal fungi groups in samples from different parts of *B. tianguii* and the samples from different habitats or from different altitude populations, but there was no significant difference in mycorrhizal fungi groups.

### Phylogenetic tree analysis

**Figure 7:**
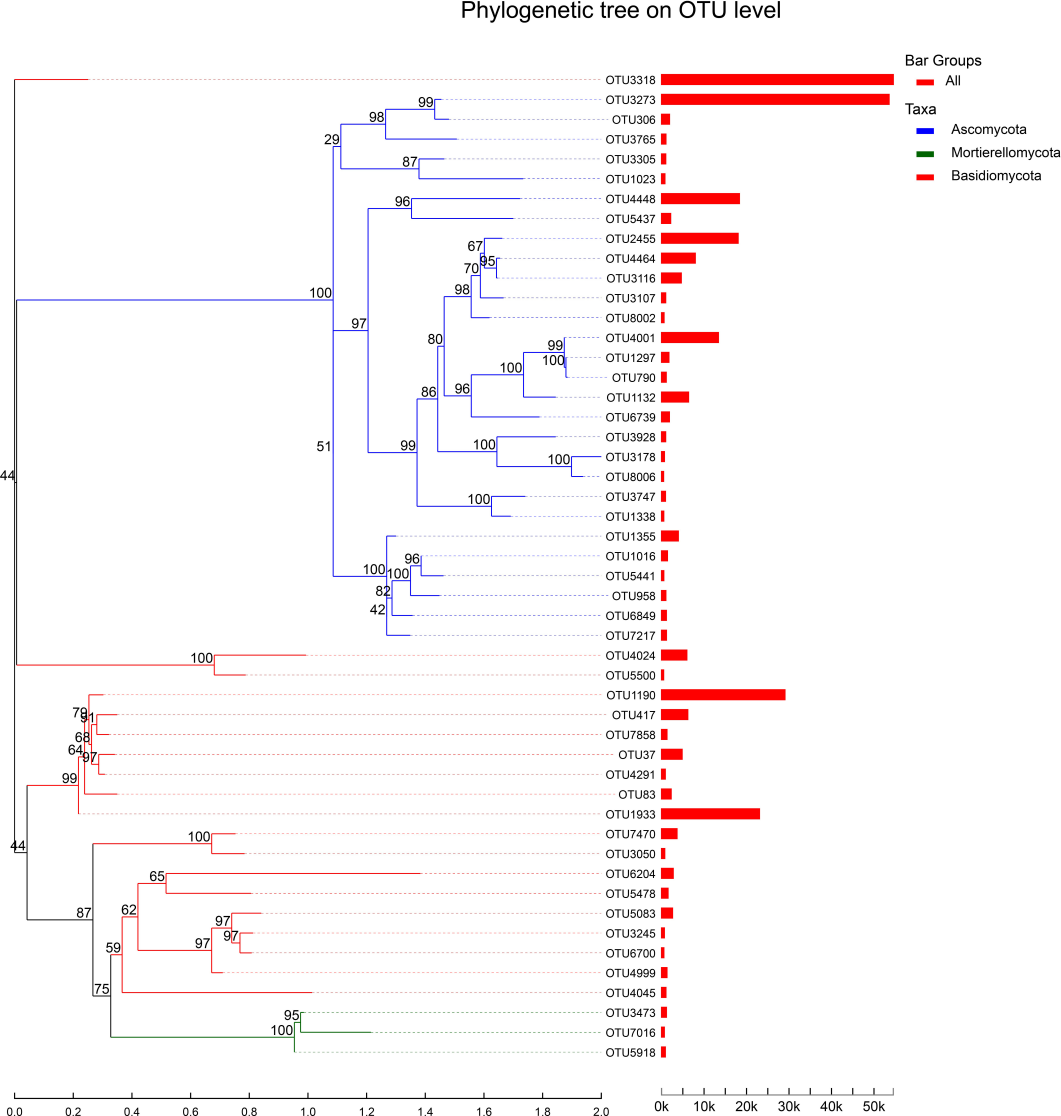
System evolution tree. Each branch represents each OTU, and the branch is colored according to the advanced Phylum level of the species. The length of the branch is the evolutionary distance between the two species, namely the degree of species difference. The column shows the proportion of Reads in different groups.

We analyzed the top 50 species of total abundance at the classification level, and the genus of all fungi detected was classified from the OTU classification level. It can be seen from the figure that the Reads proportion of OTU3318 (unclassified_o:Sebacinales) and OTU3273 (Exophiala) was the highest, which are close to 50k, followed by OTU1190 (Sebacina), OTU1933 (Sebacina), OTU4448 (Cladosporium), OTU2455 (Fusarium) and OTU4001 (unclassified_g:Trichoderma), which is also exceeded 20k. In addition, there are 35 OTUs whose reads proportion is less than 5k, and 8 OTUs whose reads proportion is at 5k-10k (**Figure 7**). The above results showed that Sebacina and Exophiala accounted for the largest proportion of mycorrhizal fungi in *B. tianguii*, which indicated that Sebacina and Exophiala are the predominance mycorrhizal fungi of *B. tianguii*.

### Co-occurrence network analysis of environmental samples of *B. tianguii*

**Figure 8:**
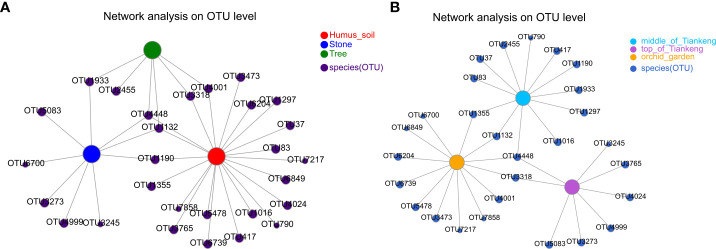
Co-occurrence network diagram. Co-occurrence relationship of species in different samples is visually displayed. Nodes in the network represent sample nodes or species nodes, and the connection between sample nodes and species nodes represents the sample containing the species. **(A)**: different habitat groups; **(B)**: different altitude groups.

The co-occurrence network analysis was performed on the environmental samples of *B. tianguii* (rhizosphere soil samples) to explore the relationship of mycorrhizal fungi in different habitats or at different altitudes (**Figure 8**). According to the different habitats of *B. tianguii*, OTUs in humus soil, stone, and tree are OTU4448 (unclassified_g:Cladosporium) and OTU1132 (unclassified_g:Trichoderma). According to the different altitude populations of *B. tianguii*, OTUs in the middle of Tiankeng, the top of Tiankeng and orchid garden is OTU4448 (unclassified_g:Cladosporium). The above results showed that the common species in different habitats and different altitudes were unclassified_g:Cladosporium, indicating that the mycorrhizal fungi of Cladosporium may be the specific symbiotic fungi of *B. tianguii*.

## Discussion

Mycorrhizal fungi have vital impacts on orchid seed germination, nutrient absorption of adult plants and population dynamics. Therefore, exploring the internal mechanism of the interaction between mycorrhizal fungi and orchids is a hot field of orchid mycorrhizal research at present (Zhao et al., 2021). However, the research on mycorrhizal fungi of *B. tianguii* is still very scarce. Studies on mycorrhizal fungi of orchids are being gradually improved, and more and more fungi are confirmed to be mycorrhizal fungi with some connection to orchids. In order to study the species of orchid mycorrhizal fungi growing in Minas Gerais state, Brazil, Nogueira et al. (Nogueira et al., 2005) isolated Ceratorhiza from the roots of *Bulbophyllum weddelii*, which is the first time that Rhizobium like fungi were isolated from orchid roots in Minas Gerais state. To identify and store important mycorrhizal fungi that can protect orchid species through symbiotic seed germination. Three mycorrhizal Rhizoctonia-like fungi were isolated from the roots of three neotropical orchid species *Gomesa crispa*, *Campylocentrum organense* and *Bulbophyllum* sp. from three different Atlantic rain forest fragments in Brazil. It was found that the isolates belonged to the genus Ceratorhiza (Pereira et al., 2005). *B. tianguii* is known to grow not only in soil and stone habitats, but also attached to the trunks of tall trees. Other researchers analyzed the fungal community composition of two orchids, *Panisea unifora* and *Bulbophyllum odoratissimum*, which grow together in forests in southern China. *P. unifora* was found to grow *Quercus yiwuensis* and *B. odoratissimum* was grown on *Pistacia weinmannifolia* trees. It was observed that Serendipitaceae (71%) and Nectriaceae (24%) were the most abundant families related to *B. odoratissimum*. Fusarium,which belongs to Nectriaceae, was found in the barks of two orchid epiphytic trees, so it cannot be ruled out that Fusarium may be important for the early stage of *B. odoratissimum* development (Pecoraro et al., 2021). The above studies have found that Fusarium and Rhizoctonia maybe a mycorrhizal fungus closely related to *B. odoratissimum* and *B.* sp., which is consistent with our findings. In addition, Ceratorhiza had been confirmed to be related to neotropical orchids *B. weddelii*, not the same to our results. This may be related to the species, distribution and adaptability of the orchid to the growing environment, and mutual selection between dominant mycorrhizal fungi and host plants (Luo et al., 2020), which needs further research and verification.

Previous studies have also confirmed that orchid mycorrhizal fungi can promote plant growth, suggesting that the mycorrhizal fungi isolated from *B. tianguii* may also have the potential function to promote plant growth and impact the host. Several different orchid mycorrhizal fungi were inoculated into *Dendrobium candidum*. It was found that Epulorhiza sp. and Rhizoctonia sp. had a good effect on the growth of *D. candidum* (Li et al., 2022). Four orchid mycorrhizal fungi isolated from the roots of wild *Paphiopedilum* (Cladosporium perangustum, Kirschsteinothelia tectonae, Phialophora sp. and Cypellophora sp.) were used to carry out bacteria seedling symbiosis test with test tube seedlings and nutrient pot seedlings of *Paphiopedilum* with leaves. The changes of growth and physiological indexes were observed. It was found that Cladosporium perangustum and Phialophora sp. increased the fresh quality of test tube seedlings. The activities of three protective enzymes and the total amount of chlorophyll were significantly affected. Kirschsteinothelia tectonae and physiophora sp. significantly improved the fresh quality increment of nutrient bowl seedlings (Chen et al., 2022). Although these observations suggest that orchid mycorrhizal fungi (OMF) is crucial in orchid microflora in orchid microbial community, little is known about the mycorrhizal fungi associated with *B. tianguii*, their interaction with host and their distribution in plants.

In the present study, the similarities and differences of mycorrhizal fungi communities in roots and rhizosphere soil of *B. tianguii* in different habitats were analyzed in order to find out possible important mycorrhizal fungi species. The analysis focused on different mycorrhizal fungi in the roots and rhizosphere soil of *B. tianguii*. OTU cluster analysis and mycorrhizal fungal community composition analysis showed that mycorrhizal fungi in roots and rhizosphere soil of *B. tianguii* mainly composed of Ascomycota and Basidiomycota. The dominant mycorrhizal fungi in roots were Ascomycota, and the dominant mycorrhizal fungi in rhizosphere soil were Basidiomycota. The dominant mycorrhizal fungi in roots and rhizomes were unclassified_g_Exophiala (37.94%) and Fusarium_concentricum (18.85%), while Sebacina_sp. (39.85%) was the dominant mycorrhizal fungi in rhizosphere soil of *B. tianguii*, indicating that Exophiala and Fusarium were the dominant genus of endophytic mycorrhizal fungi in *B. tianguii*, Sebacina_sp. is a dominant ectomycorrhizal fungus. This may be caused by the effects of different fungi on orchids or the selection of endophytic and exogenous fungi in orchids. This is consistent with other studies, which have also confirmed that Exophiala and Fusarium are common genera of orchid endophytic fungi (Tan et al., 2012).

Furthermore, the dominant mycorrhizal fungi in different habitats and altitudes of *B. tianguii* analyzed in this study are as follows: Sebacina, Exopiala in humus and stone habitat, unclassified_o:Sebacinale in tree habitat; In the middle of the Tiankeng is Sebacina, and at the top of the Tiankeng is unclassified_o: Sebacinale, the orchid garden is Exophiala. The results showed that the dominant mycorrhizal fungi of *B. tianguii* in different habitat were different, indicating that *B. tianguii* would like to choose mycorrhizal fungi that were more conducive to its survival or affected by other microorganisms. The dominant mycorrhizal fungi of *B. tianguii* at different altitudes are different, which indicates that these different mycorrhizal fungi are beneficial for *B. tianguii* to adapt to climate and soil physical and chemical properties at different altitudes. The results of phylogenetic tree and co-occurrence network analysis also indicated that the fungi belonging to Sebacina, Cladosporium, Exophiala and Fusarium might be the dominant mycorrhizal fungi symbiotic with *B. tianguii*.

Former studies have proved that orchids have a close symbiotic relationship with mycorrhizal fungi, and the dominant mycorrhizal fungi have an impact on the growth and seed germination of orchids (Qin et al., 2021; Gao et al., 2022; Li et al., 2022). Some other studies have shown that Sebacina, Exophiala and Fusarium fungi are common species in orchid mycorrhizal fungi (Tan et al., 2012; Wei et al., 2016; Fu et al., 2019), and most of them belong to Ascomycota and Basidiomycota, which is consistent with previous studies (Li T. et al., 2021; Pecoraro et al., 2017). Sebacina, Exophiala, Fusarium and Cladosporium may be related to the germination of plant seeds, plant biomass or influenced the stress resistance of plants. At present, the mycorrhizal fungi associated with *B. Tianguii* have not been reported, but some studies have found that the above mycorrhizal fungi have a certain impact on the growth of orchids. The symbiotic germination test was carried out between mycorrhizal fungi isolated from Orchidaceae and their seeds, the results showed that mycorrhizal fungi *Sebacina* sp. significantly promoted the seed germination of *Bletilla ochracea*, and the germination rate was 18.43% higher than those in the control group. This indicated that *Sebacina* sp. had a good promoting effect on seed germination of *B. striata*, which was beneficial to the protection and reproduction of *B. striata* (Chen et al., 2012). Moreover, it was also found that Sebacina vermifera can improve plant productivity and stress tolerance (Ray and Craven, 2016), Sebacina can promote *Dendrobium ofcinale* seed germination and seedling growth (Zhang et al., 2020). Fusarium is proved not only to promote the synthesis of GAs causing orchid disease, but also may form symbiotic mycelium with orchids, which is conducive to the growth of orchids (Tsavkelova, 2016; Chen et al., 2022). Attention should be paid to the interaction between Sebacina, Exophiala, Fusarium and orchids.

In the case of Cladosporium, it has positive effects on orchids as well as the possibility of causing orchid diseases. Inoculation of Cladosporium did not cause disease to the tissue culture seedlings of *Dendrobium* and *Bletilla*, and had significant growth promoting effects. Cladosporium can infect the roots of tissue culture seedlings of *D. officinale* and *B. striata*, and form a typical structure of orchid mycorrhizal fungi in root cortex cells-mycelium group. The mycorrhizal seedlings grow well and establish a mutually beneficial symbiotic system (Tang et al., 2018). The symbiotic test between *Cladosporium perangustum* isolated from the roots of wild Paphiopedilum and the test-tube seedlings of Paphiopedilum with leaf was conducted to study the changes of growth and physiological indexes. It was found that *C. perangustum* had a good inoculation effect on the test-tube seedlings. The fresh weight of the plant inoculated with *C. perangustum* increased by about 300%, and the leaf area increased by 1.11 cm^2^, which greatly improved the absorption of water in the medium. The activities of peroxidase (POD), catalase (CAT), superoxide dismutase (SOD) and total chlorophyll content in leaves were 130.3%, 1016.9%, 196.1% and 62.2% higher than those in the control, respectively, indicating that inoculation of strains was conducive to improving the protective enzyme activity and chlorophyll content in plant leaves (Chen et al., 2022). However, some studies have found that Cladosporium is related to black spot and blossom blight of *Dendrobium*, which may be an important factor leading to the wilt of precious orchids (Xiao et al., 2018; Ismail and Pauzei, 2022). Some studies have shown that Exophiala is a symbiotic mycorrhizal fungus of *Cephalanthera damasonium* and *Orchis pauciflora*, but no study has clearly pointed out that Exophiala can promote or inhibit the seed germination and growth of orchids (Pecoraro et al., 2012; Pecoraro et al., 2017). Therefore, it will be necessary for the next step to study the symbiotic relationship between the fungi of Cladosporium and *B. tianguii*.

α and β diversity analysis showed that there were significant differences in mycorrhizal fungi species in roots and rhizosphere soil of *B. tianguii*, and there were also significant differences in mycorrhizal fungi species and relative abundance of *B. tianguii* in different habitats. However, no matter from the root, rhizome and rhizosphere soil to classify or from three different habitats (humus soil, stone and tree) to classify and analyze, mycorrhizal fungi found in the root will be widely distributed in the rhizosphere soil. Species of mycorrhiza fungi of the same plant in different habitats will be similar and not completely independent of each other, which is consistent with previous research results (Waud et al., 2016).

The diversity analysis of mycorrhizal fungi in roots, rhizomes and rhizosphere soil of *B. tianguii* showed that there were significant differences in the number of mycorrhizal fungi in roots (roots, rhizomes) and rhizosphere soil, and the dominant mycorrhizal fungi were different. This difference has been confirmed in other studies that there are differences between fungal communities in plant roots and rhizosphere soil. The reason for this difference is that plant roots secrete some substances, which may regulate the types and quantities of microorganisms in soil (Wu et al., 2014; Han et al., 2021).

The diversity of mycorrhizal fungi in humus soil, stone and tree of *B. tianguii* was analyzed to verify the effects of different habitats on the number and species of mycorrhizal fungi in roots and rhizosphere soil of *B. tianguii*. The results showed that the number of mycorrhizal fungi in humus soil and stone was significantly higher than that in tree, but the number of mycorrhizal fungi in humus soil was almost equal to that in stone. The shannon index (fungal community diversity) among mycorrhizal fungi in different growth environments (humus, stone, tree) ranked as humus > stone > tree, and this difference was consistent with results found in other orchid studies (Jiang et al., 2018). The reason for these differences may be the composition of mycorrhizal fungi in orchid roots is not completely controlled by plants themselves, but the main mycorrhizal fungi still depend on the selection of orchid plants (Chen et al., 2019).

The diversity analysis of mycorrhizal fungi in the samples of different altitude populations (middle of Tiankeng, top of Tiankeng and orchid garden) of *B. tianguii* was performed to disgust the structure and diversity of mycorrhizal fungi in *B. tianguii* root and rhizosphere soil at different altitudes. The results showed that the number of mycorrhizal fungi in the middle and top of the Tiankeng was significantly lower than that in the orchid garden, but the diversity of mycorrhizal fungi in the top of the Tiankeng was significantly higher than that in the orchid garden. This was the first report on the diversity of mycorrhizal fungi of *B. tianguii* growing at different altitudes. The dominant mycorrhizal fungi in these three locations were different. This difference is consistent with previous findings, one study analyzed the diversity of root fungi of *Cremastra appendiculata* at different altitudes in Taibai Mountain Nature Reserve and Sichuan Huanglonggou. It was found that the diversity of fungi was different at different altitudes. With the increase of altitude, the number of endophytic fungi gradually decreased (Ren et al., 2021). To explore the relationship between the changes of species richness and distribution of ecorrhizal fungi (ECM) fungal community along the altitude gradient and the host distribution, the ECM fungal community in the roots of *Pinus sylvestris* at 300m and 550 - 600 m altitude in Scotland was analyzed. The results showed that the OTU richness was not related to altitude, while the ectomycorrhizal fungus *Pilderma sphaerosporum* was the most common in low altitude areas, and *Russula sardonia* was the most common in high altitude areas, indicating that with the change of altitude, the ECM fungal diversity of *P. sylvestris* did not change, but the composition of its fungal community changed, which may be closely related to the changes of soil humidity and soil temperature (Jarvis et al., 2015). The community composition of arbuscular mycorrhiza fungi (AMF) in rhizosphere soil and roots of *Spiraea pubescens* at 1515 m, 1410 m and 1305 m altitudes in Daqing Mountain, Inner Mongolia was studied. It was found that the number of OTUs of AMF was the largest at 1515 m altitude, and the highest AMF richness and diversity also appeared at the highest altitude. Altitude did not affect the species diversity of AMF, but it would affect the community structure. The reason for this phenomenon may be that these AMFs can help plants survive in a more arid and barren environment (Liu et al., 2017). And the more uneven the community distribution, and the more prominent the dominant fungi (Xu et al., 2019). The reason for this difference is that with the increase of altitude, affected by climate and environmental factors, along with changes in soil physical and chemical properties and nutrient elements, the environment is more and more unsuitable for microbial growth.

## Conclusion

In this study, high-throughput sequencing technology was used to study mycorrhizal fungal communities in roots, rhizomes and rhizosphere soil of *B. tianguii*. Species diversity analysis showed that mycorrhizal fungal communities in *B. tianguii* roots were significantly different from those in rhizomes and rhizosphere soils. The number of OTUs in rhizosphere soil and the diversity of mycorrhizal fungal communities were the highest. *B. tianguii* in different habitats and different altitudes of mycorrhizal fungi in the species and abundance are also different, the number and diversity of mycorrhizal fungi in the orchid garden and humus soil habitat are the most abundant. Species annotation, phylogenetic tree and co-occurrence network analysis indicated that the dominant mycorrhizal fungi of *B. tianguii* mainly included Sebacina, Cladosporium, Exophiala, Fusarium, belonging to Ascomycota and Basidiomycota. The results of this study showed that the symbiotic relationship between Sebacina and Cladosporium and *B. tianguii* plants needs further study. At present, there are very few studies on mycorrhizal fungi related to *B. tianguii*, and there is little knowledge about the fungal species that are closely related to *B. tianguii*. The results of this study can provide some reference for the subsequent studies on dominant mycorrhizal fungi in orchids and the protection of *B. tianguii*.

## Data availability statement

The datasets presented in this study can be found in online repositories. The names of the repository/repositories and accession number(s) can be found below: https://www.ncbi.nlm.nih.gov/, PRJNA857524.

## Author contributions

SC and XW designed the study, RZ, HQ, JT performed the experiment, JL and YL analyzed the data and drafted the manuscript, HQ, JT, RZ and SC helped in sample collection, YH helped in data analysis. All authors contributed to the article and approved the submitted version.

## Funding

This work was supported by the Guangxi Forestry Science and Technology Promotion Demonstration Project (Guilinkezi [2021] No. 28), and the Forestry Grassland Project of Central Finance of China, and the Central Guidance on Local Science and Technology Development Fund (ZY21195035), Key Laboratory of Ecology of Rare and Endangered Species and Environmental Protection (Guangxi Normal University), Ministry of Education, China (ERESEP2022Z18).

## Conflict of interest

The authors declare that the research was conducted in the absence of any commercial or financial relationships that could be construed as a potential conflict of interest.

## Publisher’s note

All claims expressed in this article are solely those of the authors and do not necessarily represent those of their affiliated organizations, or those of the publisher, the editors and the reviewers. Any product that may be evaluated in this article, or claim that may be made by its manufacturer, is not guaranteed or endorsed by the publisher.
