# Supplementary figures and images for: Structure and diversity of mycorrhizal fungi communities of different part of Bulbophyllum tianguii in three terrestrial environments
Source: Front Plant Sci. 2022 Oct 5;13:992184. doi: 10.3389/fpls.2022.992184 (PMC9579349; doi:10.3389/fpls.2022.992184)

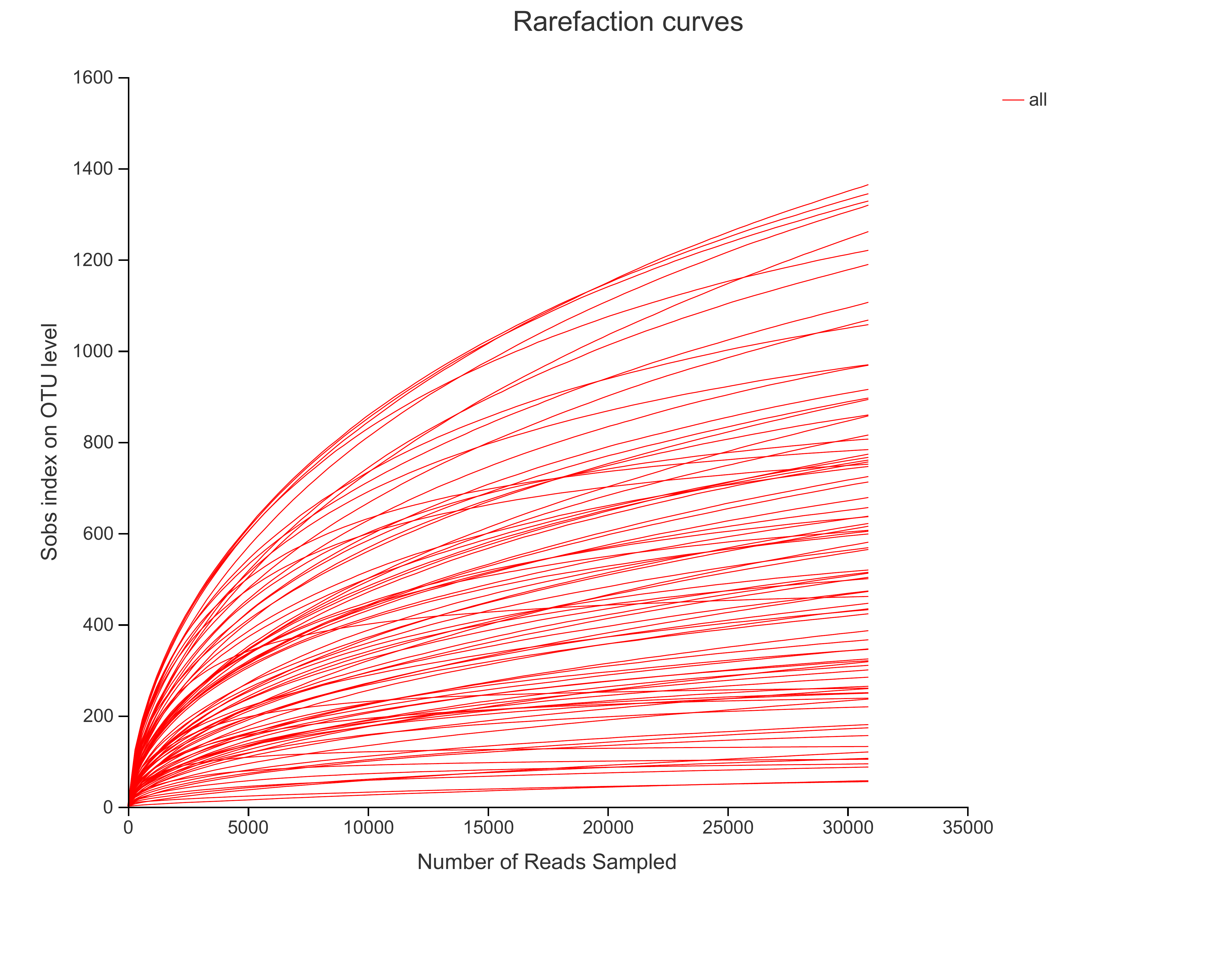

Supplement: Supplementary file 1 [file Image_1.png]
